# Inhibition in motor imagery: a novel action mode switching paradigm

**DOI:** 10.3758/s13423-016-1095-5

**Published:** 2016-06-30

**Authors:** Martina Rieger, Stephan F. Dahm, Iring Koch

**Affiliations:** 1 Medical Informatics and Technology, Institute for Psychology, UMIT - University for Health Sciences, Hall in Tirol, Austria; 2 Institute of Psychology, RWTH Aachen University, Aachen, Germany

**Keywords:** Motor imagery, Inhibition, Trial sequence effects, Global inhibition, Specific inhibition

## Abstract

Motor imagery requires that actual movements are prevented (i.e., inhibited) from execution. To investigate at what level inhibition takes place in motor imagery, we developed a novel action mode switching paradigm. Participants imagined (indicating only start and end) and executed movements from start buttons to target buttons, and we analyzed trial sequence effects. Trial sequences depended on current action mode (imagination or execution), previous action mode (pure blocks/same mode, mixed blocks/same mode, or mixed blocks/other mode), and movement sequence (action repetition, hand repetition, or hand alternation). Results provided evidence for global inhibition (indicated by switch benefits in execution-imagination (E-I)-sequences in comparison to I-I-sequences), effector-specific inhibition (indicated by hand repetition costs after an imagination trial), and target inhibition (indicated by target repetition benefits in I-I-sequences). No evidence for subthreshold motor activation or action-specific inhibition (inhibition of the movement of an effector to a specific target) was obtained. Two (global inhibition and effector-specific inhibition) of the three observed mechanisms are active inhibition mechanisms. In conclusion, motor imagery is not simply a weaker form of execution, which often is implied in views focusing on similarities between imagination and execution.

## Introduction

Executed and imagined movements exhibit many similarities: they overlap in neuronal activity (Decety, 1996; Jeannerod, 1994; Munzert, Lorey & Zentgraf, 2009 ), often have similar durations (Guillot & Collet, 2005), and often follow similar constraints (Cerritelli, Maruff, Wilson & Currie, 2000; Dahm & Rieger, 2016). However, one important difference between imagination and execution is that in imagination the movement must be prevented from actual execution, which is referred to as inhibition (Guillot, Di Rienzo, MacIntyre, Moran & Collet, 2012). It is unclear at what level inhibition takes place (Guillot et al., 2012). To investigate several forms of inhibition, we developed a new paradigm. We assumed that inhibition during motor imagery leaves after-effects by increasing activation thresholds, affecting performance of subsequently executed or imagined movements. This should be observable in sequential effects.

Currently, three inhibitory mechanisms in motor imagery are discussed (see Guillot et al., 2012, for an overview). First, because people know in advance that they will imagine and not execute a movement the need for active inhibition does not arise and inhibition is integrated into imagination. Imagination is a weaker form of execution and only subthreshold motor activation occurs (*subthreshold motor activation*) (Guillot et al., 2012). Second, all motor activity may be inhibited (*global inhibition*) (Guillot et al., 2012). Third, inhibition may be specific (Guillot et al., 2012). This may actually take two forms: inhibition may be specific for the effector used in the imagined movement (*effector-specific inhibition*) or specific for the complete action, i.e., the movement of an effector to a specific target is inhibited (*action-specific inhibition*). The different mechanisms are not necessarily mutually exclusive and may operate together or in different instances of motor imagery.

So far, inhibition in motor imagery has mainly been investigated using imaging techniques (Decety, 1996) or transcranial magnetic stimulation (Lebon, Byblow, Collet, Guillot & Stinear, 2012). In behavioral studies, inhibition cannot be observed directly. We therefore used an indirect approach to investigate inhibition in motor imagery. We adapted the experimental logic of task switching paradigms (Kiesel et al., 2010) and other paradigms (Verbruggen, Logan, Liefooghe & Vandierendonck, 2008) that are used to investigate sequential effects. In such tasks, performance in the current trial is analyzed depending on the conditions in the previous trial. It is assumed that inhibition and activation in the previous trial persist to a certain degree. This influences performance in the current trial (Anguera, Lyman, Zanto, Bollinger & Gazzaley, 2013; Kiesel et al., 2010; Koch, Gade, Schuch & Philipp, 2010; Monsell, 2003; Rieger & Gauggel, 1999; Schuch & Koch, 2003; Tipper, 2001; Verbruggen et al., 2008).

**Fig. 1:**
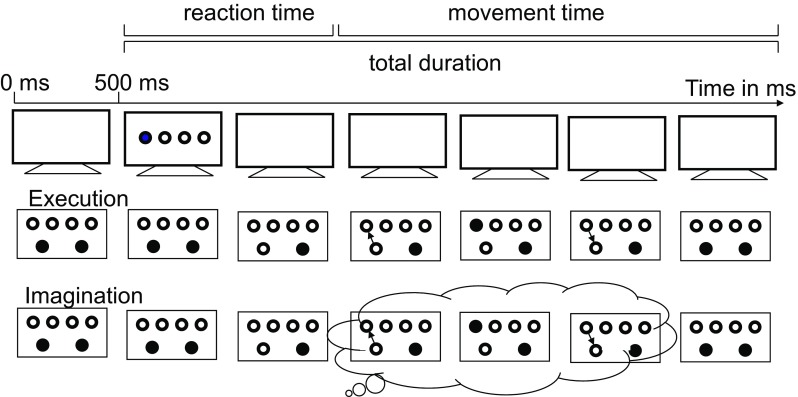
Sequence of events in an execution and an imagination trial. A trial started when participants pressed the two start buttons (start position). After an interval of 500 ms, the stimulus was presented. Upon identification of the target button, participants released the corresponding start button and the stimulus disappeared. Participants then executed or imagined moving to the target button, pressing and releasing it, and moving back to the start button. When participants arrived at the start button at the end of the (actual or imagined) movement they pressed it again. Imagination and execution were indicated by the color of the filled circle in the stimulus display

In the novel action mode switching paradigm, participants imagined (indicating only start and end) and executed movements from two start buttons (one for each hand) to one of four possible target buttons (two for each hand) upon the presentation of a visual signal (Fig. 1). Trials were presented in pure blocks (imagination or execution trials only) and mixed blocks (both imagination and execution trials). Trial sequences differed depending on *current action mode* (imagination or execution), *previous action mode* (pure blocks/same mode, mixed blocks/same mode, or mixed blocks/other mode), and the relationship between movements in two consecutive trials (*movement sequence*; complete repetition: movements with the same hand to the same target, hand repetition: movements with the same hand to the other target, or hand alternation: movements with the other hand to either target of that hand).

We expected that imagination (I) takes longer than execution (E), which is often (Calmels, Holmes, Lopez & Naman, 2006; Dahm & Rieger, 2016; Rieger, Martinez & Wenke, 2011) but not always (Dahm & Rieger, 2016; Müsseler, Wühr & Ziessler, 2014) observed with fast movements. With respect to the different inhibitory mechanisms, we can derive different scenarios as outlined below.

If motor imagery, like motor execution, results in motor activation that simply remains at subthreshold level, the same data patterns are expected regardless of whether the current action mode is imagination or execution. Thus, (a) the previous action mode should affect imagination and execution similarly, and (b) the relative difficulty of movement sequences should be similar within all combinations of previous action mode and current action mode (action mode sequences).

Global inhibition should be observable in the relative duration of different action mode sequences. In analogy to task switching, we analyzed switch costs (by comparing same action mode trials and different action mode trials in mixed blocks; Kiesel et al., 2010). If global inhibition plays a role, we expected to observe shorter durations in E (previous trial)-I (current trial)-sequences than in I-I-sequences (i.e., switch benefits), as in the latter global inhibition should take place in the previous trial, slowing down performance in the subsequent trial. In contrast, we expected to observe longer durations in I-E-sequences compared with E-E-sequences (i.e., switch costs), because global inhibition should occur in the former but not the latter.

We also analyzed mixing costs (by comparing same action mode trials in pure blocks and mixed blocks; Kiesel et al., 2010). We expected that longer durations occur in E-E-sequences in mixed blocks compared with E-E-sequences in pure blocks (i.e., mixing costs). Because in mixed blocks half of the trials consist of imagination trials, increased global inhibition may occur due to the task context. In contrast, we expected shorter durations in I-I-sequences in mixed blocks compared with I-I-sequences in pure blocks (i.e., mixing benefits), because imagination takes place less often in mixed blocks than in pure blocks, which should result in less global inhibition.

An influence of specific inhibition should be observed in the data patterns of the different movement sequences within specific action mode sequences. Executing a movement in the previous trial should result in increased activation of the used effector. This should result in a repetition benefit in E-E-sequences, i.e., faster movements in complete repetitions and hand repetitions than in hand alternations (Adam & Koch, 2014; Bertelson, 1965; Randerath, Valyear, Hood & Frey, 2015; Soetens, Boer & Hueting, 1985). If action-specific or effector-specific inhibition takes place in the previous trial, a different pattern (repetition costs instead of repetition benefits) should emerge when the movement is imagined in the previous trial (I-I-sequences and I-E-sequences). If inhibition is action-specific, repetition costs should occur in complete repetitions only. If inhibition is effector-specific, repetition costs should occur in hand repetitions and complete repetitions. Finally, in E-I-sequences, repetition costs are expected as well, because more inhibitory effort may be required in the current trial to inhibit the action or the effector which has been activated in the previous trial.

## Methods

### Participants

Psychology students (*N* = 23, 17 females, 6 males, age: *M* = 21.6 years, *SD* = 2.3 years, all right-handed, all normal or corrected to normal vision) performed the experiment for course credit. Originally 24 students participated, but 1 was excluded from analysis, because the data set was an outlier (mean durations more than 3 standard deviations above the other participants in most conditions). The number of participants was predetermined and based on past research (Dahm & Rieger, 2016). The experiment was approved by the local ethics committee and performed in accordance with the declaration of Helsinki.

### Material and apparatus

The experiment was programmed using Presentation (Version 17.2, www.neurobs.com). Participants responded on a board with six buttons (button diameter 6 cm), which was placed on a table. The two buttons close to the participants (centers 32 cm from the edge of the table; distance between centers: 30 cm) served as start buttons. The other four buttons were located behind the start buttons (15 cm away from the start buttons, at an angle of 60°) and served as target buttons. Stimuli were presented on a HPCompaqLA2206xc monitor (screen resolution: 1920 × 1080 pixels, refresh rate 5 ms) located at a viewing distance of approximately 60 cm. Stimuli consisted of four circles (2-cm diameter, 4-cm distance between centers), which were presented in the center of the screen. One of the circles was colored light or dark blue. The color indicated imagination or execution (the action–color assignment was counterbalanced between participants). The position of the filled circle indicated the target button in a spatially compatible way. The two circles on the left cued movements of the left arm and the two circles on the right cued movements of the right arm.

### Procedure

The trial procedure is depicted in Fig. 1. A trial started when participants pressed the two start buttons (start position). After an interval of 500 ms, the stimulus was presented. In both actions modes (execution and imagination), participants were asked to identify the target button and to release the corresponding start button. When the start button was released the stimulus disappeared. Instructions emphasized the details of the movement: moving to the target button, pressing the target button, releasing the target button, and moving back to the start button. In the execution condition, participants were asked to execute these elements of the movement. In the imagination condition, participants were asked to imagine performing them from a first person perspective. In both action modes, participants were asked to press the start button when they arrived at it at the end of the (actual or imagined) movement. The next trial started as soon as participants were in the start position again. Participants were instructed to imagine or execute the movements as quickly and accurately as possible and not to correct any errors.

The experiment started with three practice blocks. In the first practice block 33 execution trials, in the second practice block 33 imagination trials, and in the third practice block 65 trials consisting of both imagination and execution trials were presented. Following the practice blocks participants performed three types of experimental blocks: pure execution (3 blocks, 65 trials each), pure imagination (3 blocks, 65 trials each), and mixed blocks including imagination and execution trials (12 blocks, 65 trials each). Experimental blocks were conducted in random order. Trial order within each block was fixed and differed for each block. Trial orders were created from random sequences of trials which were modified such that all possible transitions from the previous trial to the current trial occurred equally often. This procedure resulted in twice as many hand alternation trials (as the alternate hand can move to two possible targets) than complete repetition and hand repetition trials. All other conditions were equally distributed. Participants had the opportunity to take a break after each block. The whole experiment took approximately 1.5 hours. An experimenter was present the whole time to make sure participants followed the instructions and to answer any questions.

### Data analysis

Trials were not included in the analysis if: (a) participants reacted before the stimulus appeared (anticipations) or (b) participants did not press a target button after an execution stimulus or pressed a target button after an imagination stimulus (together: 2.4 % in imagination, 2.9 % in execution). Errors (movements to a wrong target button) were included in the analysis, because they cannot be determined in imagination trials. We report reaction time (duration from stimulus presentation to the release of the start button, RT) and movement time (duration from release of the start button to the press of the start button at the end of the movement, MT).

Repeated measures analysis of variance (ANOVAs) with the within factors current action mode (imagination or execution), previous action mode (pure blocks/same mode, mixed blocks/same mode, or mixed blocks/other mode), and movement sequence (complete repetition, hand repetition, or hand alternation) were performed on RT and MT. If Mauchly’s test indicated that the assumption of sphericity was violated, we report Greenhouse-Geisser corrected degrees of freedom and *p* values, and Greenhouse-Geisser’ε. If the relevant main effects and/or interactions were significant, we conducted post-hoc tests in accordance with the hypotheses outlined above. The significance level for post hoc tests was corrected using the Holm-Šídák procedure. Where appropriate minimum (*p*_min_), maximum (*p*_max_), or exact *p* values are reported. *M*_*diff*_ indicates the difference between two conditions.

## Results

**Fig. 2:**
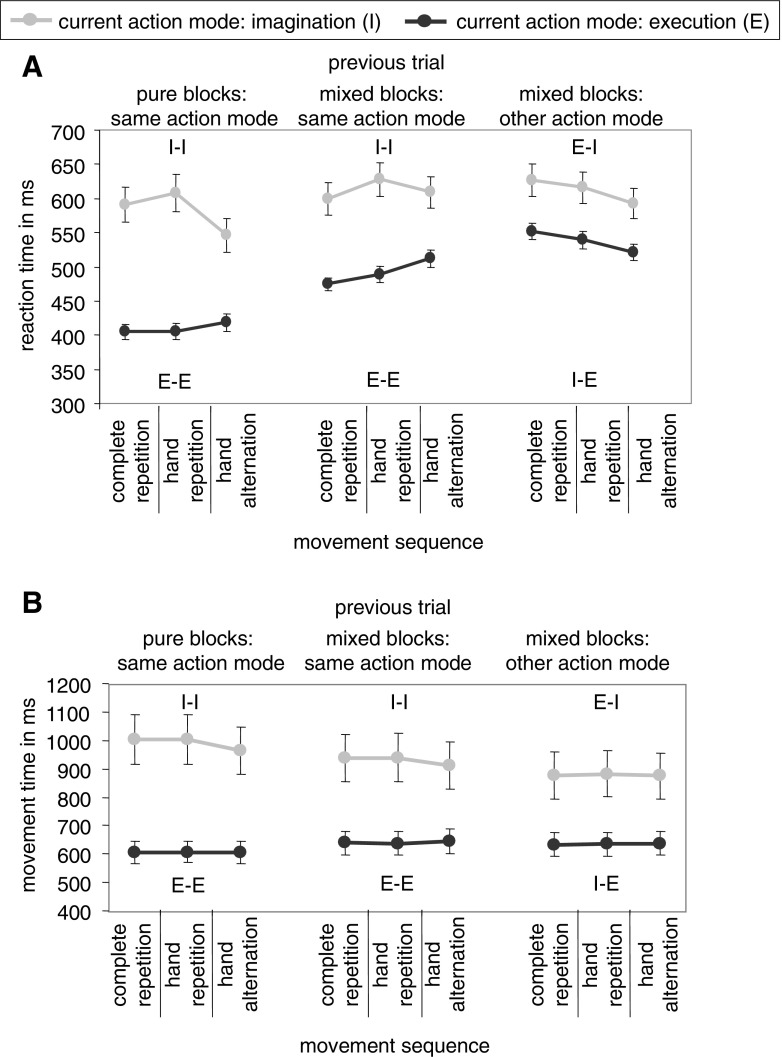
Means and standard errors of reaction times (**A**) and movement times (**B**), depending on current action mode (imagination or execution), previous action mode (pure blocks/same mode, mixed blocks/same mode, or mixed blocks/other mode), and movement sequence (complete repetition, hand repetition, or hand alternation). Letters in the figure denote the action mode sequence (I = imagination, E = execution). The first letter refers to the previous trial, the second letter to the current trial

**Fig. 3:**
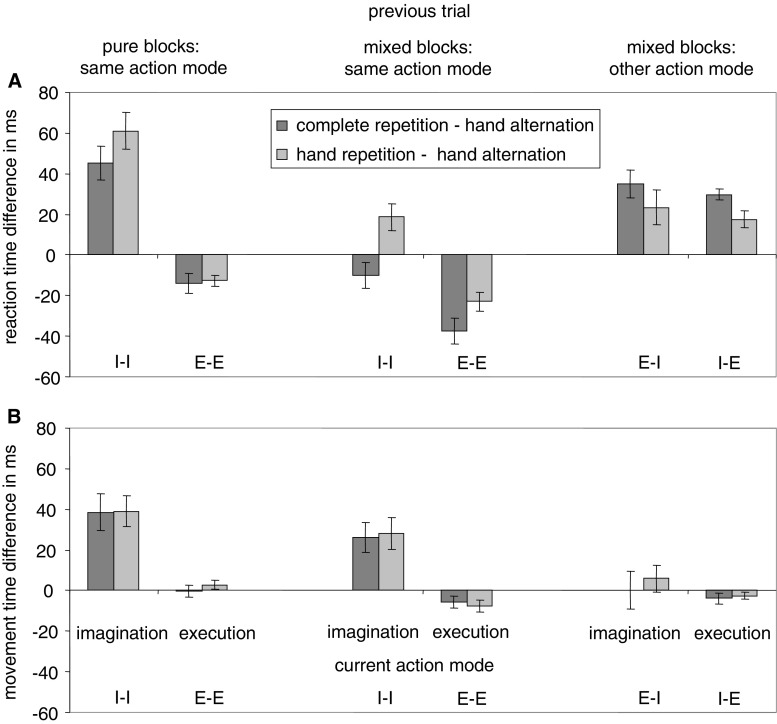
Means and standard errors of differences between movement sequences (complete repetitions minus hand alternations and hand repetitions minus hand alternations) in reaction time (**A**) and movement time (**B**), depending on current action mode (imagination or execution) and previous action mode (pure blocks/same mode, mixed blocks/same mode, or mixed blocks/other mode). Letters in the figure denote the action mode sequence (I = imagination, E = execution). The first letter refers to the previous trial, the second letter to the current trial. Positive values denote costs, negative values benefits

Means and standard errors of RTs and MTs are shown in Fig. 2. Means and standard errors of differences between movement sequences (complete repetitions minus hand alternations and hand repetitions minus hand alternations) in RT and MT are shown in Fig. 3. The results of the ANOVAs are recorded in Table 1.

**Table 1:** Results of the ANOVAs with the factors current action mode (imagination or execution), previous action mode (pure blocks/same mode, mixed blocks/same mode, or mixed blocks/other mode), and movement sequence (complete repetition, hand repetition, or hand alternation) on reaction time and movement time

	df, ε	F	*p*	η_p_^2^
*Reaction time*				
Current action mode	1, 22	32.4	< .001	.60
Previous action mode	1.5, 32, ε = .73	48.6	.001	.89
Movement sequence	2, 44	10.7	< .001	.33
Current action mode * previous action mode	1.3, 28.9, ε = .66	19.4	< .001	.47
Current action mode * movement sequence	1.4, 31.7, ε = .72	34.8	< .001	.61
Previous action mode * movement sequence	4, 88	25.7	< .001	.54
Current action mode * previous action mode * movement sequence	4, 88	15.1	< .001	.41
*Movement time*				
Current action mode	1, 22	21.4	< .001	.49
Previous action mode	1.2, 27.4, ε = .62	5.73	.018	.21
Movement sequence	1.5, 32.1, ε = .73	8.82	.002	.29
Current action mode * previous action mode	1.5, 33.2, ε = .75	22.5	< .001	.51
Current action mode * movement sequence	2, 44	16.8	< .001	.43
Previous action mode * movement sequence	2.6, 57, ε = .65	5.1	.005	.19
Current action mode * previous action mode * movement sequence	4, 88	4.7	.002	.18

ε = Greenhouse-Geisser’ε

### Reaction times

The significant main effect of current action mode indicated that RTs were longer in imagination (*M* = 602 ms) than in execution (*M* = 480 ms). The significant interaction between current action mode and previous action mode modified the significant main effect of previous action mode. Switch costs (different action mode vs. same action mode in mixed blocks) were observed when the current action mode was execution, i.e., in I-E-sequences (*M* = 538 ms) compared with E-E-sequences (*M* = 492 ms, *M*_*diff*_ = 46 ms*, p* < .001), but not when the current action mode was imagination, i.e. in E-I-sequences (*M* = 612 ms) in comparison to I-I-sequences, (*M* = 612 ms, *M*_*diff*_ = .01 ms, *p* = .99). Mixing costs (same action mode in mixed blocks vs., same action mode in pure blocks) were observed in both E-E-sequences (*M*_*diff*_ = 82 ms, *p* < .001, pure blocks: *M* = 410 ms) and I-I-sequences (*M*_*diff*_ = 30 ms, *p* = .038, pure blocks: *M* = 582 ms).

To analyze the three-way interaction, we looked at the durations of movement sequences (complete repetitions, hand repetitions, and hand alternations) within action mode sequences. In E-E-sequences, both in pure and mixed blocks, RTs were significantly shorter in complete repetitions and hand repetitions than in hand alternations (*p*_max_ = .01). RTs in complete repetitions were significantly shorter than in hand repetitions in mixed blocks (*p* = .016) but not in pure blocks (*p* = .71). In all other sequences, a different pattern emerged. In I-I-sequences, both in pure and mixed blocks, RTs in hand repetitions were significantly longer than in hand alternations (*p*_max_ = .009) and complete repetitions (*p*_max_ = .006). RTs in complete repetitions were significantly longer than in hand alternations in pure blocks (*p* <.001) and not significantly different from hand alternations in mixed blocks (*p* = .13). In E-I-sequences, RTs in hand alternations were significantly shorter than in hand repetitions and complete repetitions (*p*_max_ = .011). RTs in hand repetitions and complete repetitions did not significantly differ from each other (*p* = .066). In I-E-sequences RTs were significantly shorter in hand alternations than in hand repetitions and complete repetitions (*p*_max_<.001) and RTs in hand repetitions were significantly shorter than in complete repetitions (*p* = .002).

### Movement times

The significant main effect of current action mode indicated that MTs were longer in Imagination (*M* = 933 ms) than in execution (*M* = 627 ms). The significant interaction between current action mode and previous action mode modified the significant main effect of previous action mode. Switch costs were not observed when the current action mode was execution, i.e., in I-E-sequences (*M* = 635 ms) compared with E-E-sequences (*M* = 641 ms, *M*_*diff*_ = 6 ms, *p* < .07). When the current action mode was imagination, i.e., in E-I-sequences (*M* = 878 ms) compared with I-I-sequences (*M* = 930 ms) *switch benefits* were observed (*M*_*diff*_ = 51 ms, *p* = .003). Mixing costs were observed in E-E-sequences (*M*_*diff*_ = 36 ms, *p* = .002, pure blocks: *M* = 605 ms) but in I-I-sequences a *mixing benefit* was observed (*M*_*diff*_ = 62 ms, *p* = 0.006, pure blocks: *M* = 991 ms).

To analyze the three-way interaction, we again analyzed the durations of movement sequences within action mode sequences. No significant effects were observed in most conditions. Only in I-I-sequences, both in pure and mixed blocks, MTs were significantly shorter in hand alternations than in hand repetitions and complete repetitions (*p*_max_ = .002). Complete repetitions and hand repetitions did not significantly differ from each other (*p*_min_ = .62).

## Discussion

To investigate at what level inhibition takes place in motor imagery, we developed a novel action mode switching paradigm. Participants executed or imagined movements from starting positions to target positions. We analyzed RTs and MTs as a function of trial sequence. Trial sequences differed depending on current action mode (imagination or execution), previous action mode (pure blocks/same mode, mixed blocks/same mode, or mixed blocks/other mode), and movement sequence (complete repetition, hand repetition, or hand alternation).

As expected, imagination was slower than execution. Most likely, alternations between execution (indicating the start), inhibition (imagined movement), and execution (indicating the end) resulted in longer durations in imagination than in execution (Dahm & Rieger, 2016). Evidence for subthreshold motor activation during imagination was not observed, because the previous action mode affected imagination and execution in different ways and the relative difficulty of movement sequences differed in different action mode sequences.

Evidence for global inhibition was provided by switch benefits in E-I-sequences compared to I-I-sequences in MT. This may reflect lower global inhibition after an execution trial compared to an imagination trial. In RT benefits from lower global inhibition and costs due to switching between action modes might have cancelled each other out. Switch costs in I-E-sequences compared with E-E-sequences in RT most likely result from the requirement to switch between action modes but global inhibition also may contribute to this effect.

Mixing costs in RT, both in E-E-sequences and I-I-sequences, might reflect increased task difficulty due to more conditions in mixed blocks. In MT mixing benefits in I-I-sequences might indicate that global inhibition is lower in a context in which participants sometimes have to execute movements. Mixing costs in E-E sequences in MT might indicate that global inhibition takes place when participants have to execute a movement in a context in which movements are sometimes imagined. However, mixing costs/benefits are less directly related to global inhibition than switch costs/benefits, because the previous action mode is the same.

Data patterns within action mode sequences mainly varied in RT. The few effects in MT were consistent with the results from RT. Data patterns were markedly different between E-E-sequences and all other sequences. In E-E-sequences benefits occurred in complete repetitions and hand repetitions (Bertelson, 1965). In all other sequences repetition costs occurred. In I-I-sequences and I-E-sequences repetition costs indicate that effector-specific inhibition occurs during imagination in the previous trial, resulting in costs when the same effector is used in the subsequent trial. In I-I-sequences, complete repetitions were performed faster than hand repetitions. However, complete repetitions were still slower than (pure blocks) or not significantly different from (mixed blocks) hand alternations. Most likely, hand repetition costs and target repetition benefits (see below) both affected complete repetitions. Thus, inhibition is effector-specific but not action-specific. It does not include the movement parameters (i.e., the direction or the target) associated with the effector. This would have resulted in higher costs in complete repetitions than hand repetitions. In E-I-sequences, we also observed repetition costs. Here the use of a specific effector in the previous trial probably resulted in facilitation of this effector (Soetens et al., 1985) and more inhibitory effort is required when a movement with the same effector is imagined in the current trial.

As outlined, evidence for subthreshold motor activation was not observed. There is another way how inhibition may be integrated in motor imagery. In I-I-sequences, target repetition benefits and in I-E-sequences target repetition costs occurred. When a target location is encountered in an imagination context in the previous trial, the location might become associated with a code indicating that no actual movement should take place (*target inhibition*). This code may be retrieved in the current trial and facilitate imagination and interfere with execution of any movement to that target (Logan, 1990; Mayr & Bryck, 2005). Target repetition significantly influenced RTs when the movement was imagined in the previous trial, but not when it was executed. Possibly, targets receive more attention in imagination because of interference between one’s perceived position and one’s imagined position (Campos, Siegle, Mohler, Bülthoff & Loomis, 2009) or because participants focus more on the details of a movement in imagination (Calmels et al., 2006; Rieger et al., 2011).

Can a similar mechanism also explain the effects we attributed to effector-specific inhibition? There is an ongoing debate about whether sequential effects are due to active inhibition or due to other mechanisms (Koch et al., 2010; MacLeod, Dodd, Sheard, Wilson & Bibi, 2003; Tipper, 2001), which also concerns sequential effects in response inhibition tasks (Verbruggen et al., 2008). It is argued that when response inhibition is required in a particular trial, the stimulus becomes associated with that inhibition. The next time the stimulus repeats this inhibition is retrieved, resulting in longer durations (Rieger & Gauggel, 1999; Verbruggen et al., 2008). However, such a mechanism cannot explain the observed hand repetition costs when the target did not repeat. Interference between using the same hand to move to different locations in the current trial and the previous trial also cannot explain hand repetition costs after imagination in the previous trial, as hand repetition benefits were observed in E-E-sequences.

In conclusion, we developed a novel experimental paradigm to investigate inhibition in motor imagery. We observed evidence for global inhibition, effector-specific inhibition, and target inhibition. No evidence for subthreshold motor activation or action-specific inhibition was obtained. Importantly, two (global inhibition and effector-specific inhibition) of the three observed mechanisms are active inhibition mechanisms. Data show that motor imagery is not simply a weaker form of execution, which often is implied in views focusing on similarities between imagination and execution. Future studies should investigate the flexibility and the time course of the different inhibitory mechanisms in motor imagery, why RT and MT differ in global inhibition, and how inhibitory efficiency in the previous trial (for instance indicated by subthreshold muscle activity) affects sequential effects observed in the current trial.
